# S100A6 participates in initiation of autoimmune encephalitis and is under epigenetic control

**DOI:** 10.1002/brb3.2897

**Published:** 2023-02-07

**Authors:** Chih‐Hsiang Lin, Sung‐Chou Li, Ming‐Hong Lin, Chen‐Jui Ho, Yan‐Ting Lu, Yuyu Lin, Pei‐Hsien Lin, Kuo‐Wang Tsai, Meng‐Han Tsai

**Affiliations:** ^1^ Department of Neurology Kaohsiung Chang Gung Memorial Hospital and Chang Gung University College of Medicine Kaohsiung Taiwan; ^2^ Genomics and Proteomics Core Laboratory Kaohsiung Chang Gung Memorial Hospital and Chang Gung University College of Medicine Kaohsiung Taiwan; ^3^ Department of Microbiology and Immunology, School of Medicine College of Medicine Kaohsiung Medical University Kaohsiung Taiwan; ^4^ Research Center for Environmental Medicine, School of Medicine College of Medicine Kaohsiung Medical University Kaohsiung Taiwan; ^5^ Department of Research Taipei Tzu Chi Hospital, Buddhist Tzu Chi Medical Foundation New Taipei Taiwan; ^6^ Medical School College of Medicine Chang Gung University Taoyuan Taiwan

**Keywords:** autoimmune encephalitis, B lymphocyte infiltration, DNA methylation, leukocyte‐endothelial adhesion, S100A6

## Abstract

**Introduction:**

Autoimmune encephalitis (AE) is caused by autoantibodies attacking neuronal cell surface antigens and/or synaptic antigens. We previously demonstrated that *S100A6* was hypomethylated in patients with AE and that it promoted B lymphocyte infiltration through the simulated blood–brain barrier (BBB). In this study, we focused on the epigenetic regulation of *S100A6*, the process by which S100A6 affects B lymphocyte infiltration, and the therapeutic potential of S100A6 antibodies.

**Methods:**

We enrolled and collected serum from 10 patients with AE and 10 healthy control (HC) subjects. Promoter methylation and 5‐azacytidine treatment assays were conducted to observe the methylation process of *S100A6*. The effect of S100A6 on B lymphocytes was analyzed using an adhesion assay and leukocyte transendothelial migration (LTEM) assay. A LTEM assay was also used to compare the effects of the serum of HCs, serum of AE patients, S100A6 recombinant protein, and S100A6 antibodies on B lymphocytes.

**Result:**

The promoter methylation and 5‐azacytidine treatment assays confirmed that *S100A6* was regulated by DNA methylation. The adhesion study demonstrated that the addition of S100A6 enhanced adhesion between B lymphocytes and a BBB endothelial cell line in a concentration‐dependent manner. The LTEM assay showed that the serum of AE patients, as well as S100A6, promoted B lymphocyte infiltration and that this effect could be attenuated by S100A6 antibodies.

**Conclusion:**

We clarified that S100A6 was under epigenetic regulation in patients with AE and that it helped B lymphocytes to adhere to and infiltrate the BBB endothelial layer, which could be counteracted by S100A6 antibodies. Therefore, the methylation profile of *S100A6* could be a marker of the activity of AE, and countering the effect of S100A6 may be a potential treatment target for AE.

## Graphical abstract

We demonstrated that S100A6 participated in promoting B lymphocyte infiltration through the blood‐brain barrier in patients with autoimmune encephalitis. This promoted infiltration effect could be attenuated by the S100A6 antibody. Further investigation of the effect of S100A6 can improve our understanding of the pathogenesis of autoimmune encephalitis and serve as a potential therapeutic target.

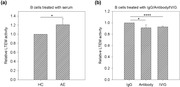

## 1 INTRODUCTION

Autoimmune encephalitis (AE) is caused by autoantibodies that attack the neuronal cell surface or synaptic antigens (Leypoldte et al., 2015). The disease usually presents with prodrome symptoms such as fever, headache, or upper respiratory infection that resemble a viral infection (Jang et al., 2020). Therefore, it is easily confused with infectious encephalitis due to the similar initial presentation (Lin et al., 2019), and it may be harmful if not treated promptly. Although the diagnosis of AE is aided by expert opinion‐based consensus criteria (Graus et al., 2016), autoimmune tests, and/or the response to immunotherapy (Jang et al., 2020; Zuliani, 2012), there are still limitations with these methods, and the diagnosis can still be challenging (Abboud et al., 2017; Graus et al., 2016). Consequently, there is an unmet need for biomarkers to predict the presence of AE or monitor its activity.

How aberrant autoimmunity develops in AE is still unknown. It is presumed, similar to other autoimmune diseases, that a complex interaction of genetic background, environmental exposure, and epigenetic process all contribute to the development of AE (Kim et al., 2014). The epigenetic process is defined as the influence of genetic activity without changing the DNA sequence, including DNA methylation, histone modification, nucleosome positioning, and microRNAs (Funes et al., 2021). In multiple sclerosis, an archetypal autoimmune disease of the central nervous system (CNS), the DNA methylation pattern has been reported to differ among different blood cells (Kiselev et al., 2021). To the best of our knowledge, only a few studies have investigated the epigenetic process of AE. We previously explored the role of DNA methylation in AE using a genome‐wide methylation microarray and identified that the coding region for *S100A6* was hypomethylated in AE patients, compared with healthy controls (HCs; Tsai et al., 2019). In the present study, we further investigated the function of S100A6 on endothelial cells and the therapeutic potential of targeting S100A6 in patients with AE. Altered expressions of genes could serve as a potential marker to aid in the diagnosis of AE or develop new therapies for AE. Since S100A6 promotes the infiltration of B lymphocytes through the blood–brain barrier (BBB; Tsai et al., 2019), we hypothesized that this effect could be counteracted by S100A6 antibodies.

## 2 MATERIALS AND METHODS

### 2.1 Study subject enrollment

We enrolled adult subjects with AE who were treated at Kaohsiung Chang Gung Memorial Hospital, Taiwan. All subjects gave informed consent for inclusion before they participated in the study. The study was conducted in accordance with the Declaration of Helsinki, and the protocol was approved by the Institutional Review Board of Kaohsiung Chang Gung Memorial Hospital (IRB approval No. 202000505B0). The patients were diagnosed with AE according to experts’ consensus (Graus et al., 2016) as follows:

1. A subacute onset of neurological deficit including working memory deficits, change of mentality status, or psychiatric symptoms that progressed for fewer than 3 months.
2. One of the following must be presented:
3. New focal neurological findings of the CNS.
4. New‐onset seizures or seizures cannot be attributed to a previously known seizure disorder.
5. The white blood cell count of cerebrospinal fluid is more than 5 cells/mm^3^.
6. Encephalitis is suggested by magnetic resonance imaging.
7. Reasonable exclusion of alternative causes.

In addition to AE subjects, volunteer adult subjects without a history of neurological or systemic autoimmune disorders were also enrolled to form the HC set.

All patients who fulfilled these criteria were tested for the presence of antibodies against neuronal surface antigens, including anti‐N‐methyl‐D‐aspartate(NMDA), alpha‐amino‐3‐hydroxy‐5‐methyl‐4isoxazolepropionic acid(AMPA), leuine‐rich, glioma inactivated 1(LGI1), contactin‐associated protein‐like 2(CASPR2), and gamma‐aminobutyric acid(GABA)‐B receptors, using a commercial kit (Autoimmune Encephalitis Mosaic 6 Assay, EUROIMMUN). We enrolled patients who fulfilled the diagnostic criteria for AE and tested positive for antibodies against neuronal surface antigens. Patients with negative assay results but who fulfilled the diagnostic criteria for AE were enrolled only if they responded to immunotherapy.

### 2.2 Serum enrichment, ELISA, and cell culture

Total blood was collected from the enrolled AE patients and HC subjects, and the serum was enriched and stored at −80°C before assay. We conducted enzyme‐linked immunosorbent assay (ELISA) to measure the protein level of S100A6 in subject serum samples by referring to the protocol of the S100A6 ELISA kit (ARG82251, Arigo biolaboratories). We also cultured one B cell line (Toledo, CRL‐2631, ATCC) one neutrophil‐like cell line (HL‐60, 60027, BCRC), one BBB endothelial cell line (human cardiac microvascular endothelial cells(hCMEC)/D3, Millipore Cat. #SCC066), and one human embryonic kidney cell line (293T). These cells were cultured according to the manufacturer's instructions. Briefly, Toledo cells were cultured in the following medium: 90% Roswell Park Memorial Institute(RPMI) 1640 medium with 2 mM l‐glutamine adjusted to contain 1.5 g/L sodium bicarbonate, 4.5 g/L glucose, 10 mM N‐2‐hydroxethylpiperazine‐N‐2‐ethane sulfonic acid(HEPES), and 1.0 mM sodium pyruvate + 10% fetal bovine serum. hCMECs were cultured with vascular endothelial growth factor(VEGF)‐free EndoGRO‐MV culture medium. HL‐60 was differentiated into the neutrophil‐like cell by the induction of 1.3% dimethyl sulfoxide(DMSO)(Sigma–Aldrich).

### 2.3 Promoter methylation assay

We conducted a promoter methylation assay as previously described (Yang et al., 2016). In brief, the putative promoter region of the *S100A6* gene was polymerase chain reaction (PCR)‐amplified, followed by digestion with the restriction enzyme HindIII and cloning into the pGL4.21 luciferase expression vector (Promega). The vector was then subjected to in vitro methylation using M. SssI, M. Hhal, and M. Hpall methyltransferase enzymes (Invitrogen), which recognize the sequence patterns CG, CGCG, and CCGG, respectively. These three enzymes catalyze in vitro cytosine methylation at the recognized sequence pattern. Finally, a luciferase assay was conducted with the 293T cells using a Dual‐Glo luciferase reporter assay system kit (Promega) 24 h after transfection.

### 2.4 5‐azacytidine treatment and qPCR assay

After differentiating into neutrophil morphology, HL‐60 cells were treated with 5‐azacytidine (Sigma–Aldrich) with the dosages of 5, 10 μM/L for 96 h by referring to a previous study (Wang et al., 2010). Then, the treated and untreated HL‐60 cells were subjected to RNA extraction and qPCR assay by referring to our previous study (Tsai et al., 2019). The forward and reverse primers for *S100A6* were TTCCACAAGTACTCCGGCA and ACCTCCTGGTCCTTGTT, respectively. The forward and reverse primers for S18 (internal control) were individually GTAACCCGTTGAACCCCATT and CCATCCAATCGGTAGTAGCG.

### 2.5 Endothelial cell adhesion assay

We conducted an endothelial cell adhesion assay as previously described (Zhang et al., 2021). In brief, we first treated Toledo cells with different concentrations of S100A6 recombinant protein for 24 h, including 0 pg/ml, 11pg/ml (20% of the mean concentration in eight AE patients), or 22pg/ml (40% of the mean concentration in eight AE patients). The Toledo cells were also treated with 20% pooled serum of either HC control or AE patients. Then, the treated Toledo cells were subjected to staining with 2',7'‐Bis(2‐carboxyethyl)‐5(6)‐carboxyfluorescein(BCECF) dye (0.5 mM, carrying green fluorescence) for 30 min. In addition, 3*10^5^ hCMEC cells were seeded in a 12‐well plate for 24 h. After staining, 1*10^5^ Toledo cells were cultured with hCMECs by placing the Toledo cells on a plate in which the hCMECs had been seeded 24 h in advance. After co‐culture for 1 h, the plates were washed with Hank's Balanced Salt Solution(HBSS) buffer to remove unadhered Toledo cells. Finally, the plates were examined with a fluorescence reader (FLx800, BioTek) to record fluorescence intensities. In addition to fluorescence intensity, we also used a camera to record the number of cells emitting green fluorescence.

### 2.6 In vitro leukocyte transendothelial migration (LTEM) assay

We examined the transendothelial infiltration ability of Toledo cells using an LTEM assay as previously described (Huang et al., 2018). To mimic the BBB endothelial layer, 2*10^5^ hCMECs were first seeded into gelatin‐coated hanging inserts (Merck). After 24 h of culture, the inserts were then placed into 24‐well culture plates. Two separate experiments were conducted. First, the Toledo cells were treated with either 20% HC serum or 20% AE serum to observe the impact of different serums on the Toledo cells. The second experiment was to observe if treatment targeting the S100A6 protein could alter the effect of disease on the Toledo cells. The Toledo cells were first treated with 20% AE serum followed by either IgG control (UB276978, Thermo), S100A6 antibodies (MA5‐32511, Invitrogen), or human immunoglobulin (IVIG; 3740501374, TBSF, Taiwan). The dosages of Immunoglobin‐G(IgG) control, S100A6 antibody, and IVIG were all 11 pg/ml, which is almost equal to 20% of the mean concentration of S100A6 serum protein (54.99*0.2 = 11.00) in the 10 AE patients. Human immunoglobulin (IVIG) is an effective first‐line immunotherapy for patients with AE (Trewin et al., 2022). Then, 1*10^5^ Toledo cells were placed in the inserts for 2 h to penetrate the BBB endothelial layer. The Toledo cells that had migrated onto the culture plate were collected, stained with CD19‐FITC (BD Biosciences), and quantified using LSRII flow cytometry (BD Biosciences).

## 3 RESULTS

### 3.1 Subjects information

A total of 10 (six male and four female) AE patients and 10 (six male and four female) HC subjects were enrolled, and their clinical characteristics are shown in Table 1. There was no significant difference in average age between the HC subjects and AE patients (44.5 ± 12.3 vs 48.0 ± 27.4 years; *t*‐test *p*‐value of .97). There was also no significant difference in sex between the two groups (chi‐square *p*‐value of .46).

**TABLE 1:** The demographic characteristics and clinical symptoms of the enrolled subjects

	Healthy controls (*n* = 10)	Autoimmune encephalitis patients (*n* = 10)	*p*‐value*
Age (years)	44.5 ± 25.3	48.0 ± 27.4	.97
Sex	50% (male)	60% (male)	.46
Clinical symptoms		Short‐term memory loss (40%)	
		Altered mental status (80%)	
		Psychiatric symptoms (20%)	
		Seizures (80%)	
		Involuntary movement (60%)	
		MRI features suggestive of	
		encephalitis (40%)	
		CSF pleocytosis (20%)	
		New focal CNS findings (60%)	

Abbreviations: CNS, central nervous system; CSF, cerebrospinal fluid; MRI, magnetic resonance imaging.

* The *t*‐test was used for age, and the chi‐square test was used for sex.

### 3.2 S100A6 was regulated by DNA methylation

**FIGURE 1:**
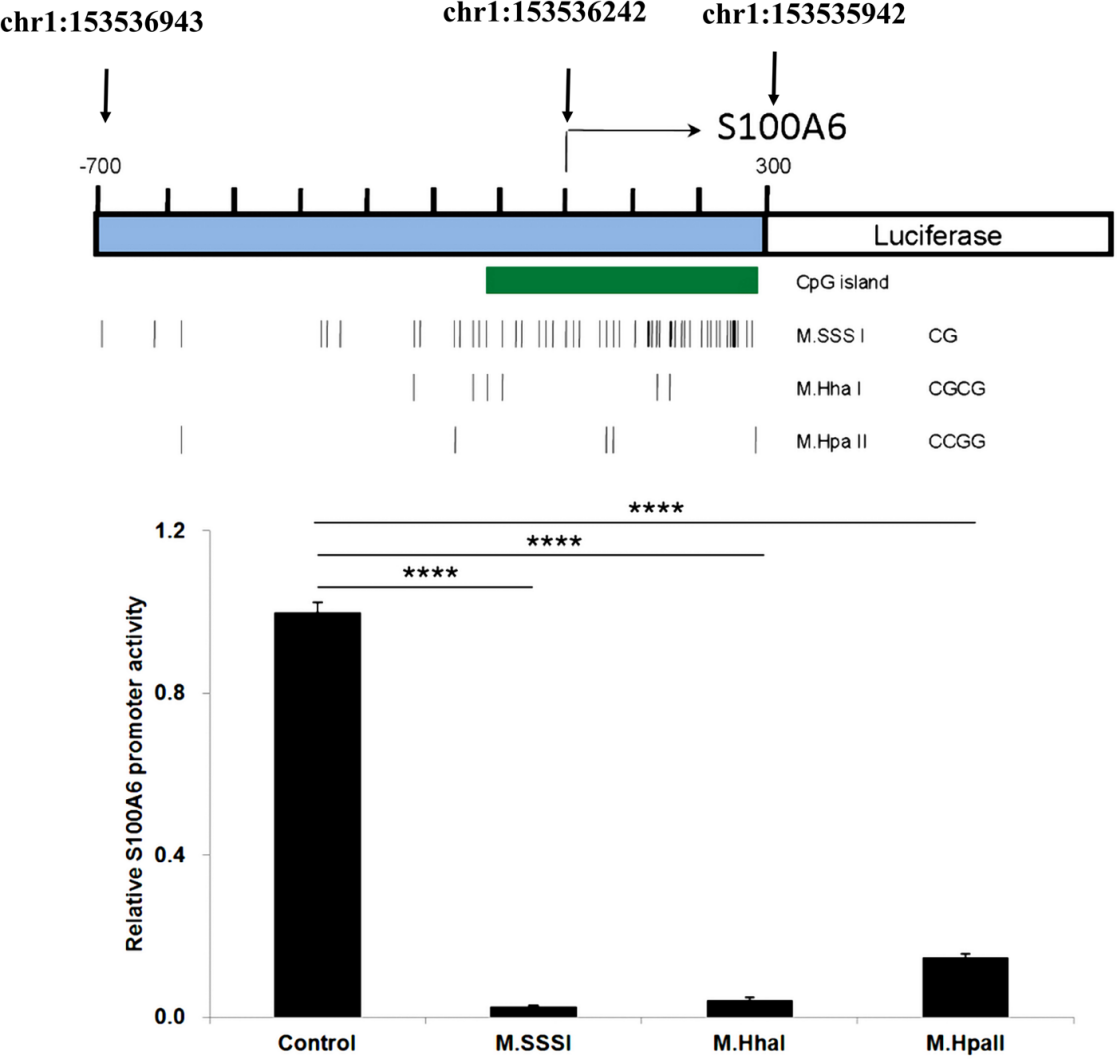
The results of promoter methylation. The putative promoter fragment (chromosome 1:153535942‐153536943) of the upstream region of S100A6 was inserted into the pGL4.21 vector (which lacks a promoter). The PGL4.21‐S100A6 promoter construct was methylated by M.SssI, M. HhaI, or M. HpaII. After in vitro methylation, the efficiency of each methylating plasmid was confirmed through restriction enzyme digestion (Supplementary Figure S1). The construct was further expressed in 293T cells to measure the activity of luciferase. The vertical lines denote the locations of CG, CGCG, and CCGG motifs, which were the targets of M. SssI, M. Hhal, and M. Hpall methyltransferase enzymes, respectively. After treatment with the methyltransferase enzyme, the promoter regions resulted in an increase in luciferase activity. Data were presented as mean ± SD. *, **, ***, and **** denote *p*‐values of < .05, .01, .001, and .0001, respectively. This applies to all figures.

In our previous study, we observed that hypomethylation at the promoter regions of *S100A* resulted in higher gene expression levels in the white blood cells of AE patients (Tsai et al., 2019). To further investigate whether *S100A6* is indeed regulated by the methylation of these promoters, we conducted a promoter methylation assay combining promoter cloning and in vitro DNA methylation. As shown in Figure 1, we cloned the promoter region of *S100A6* and ligated it with the luciferase gene. As they recognize different target sites, M. SssI M. HhaI, and M. HpaII caused different extents of DNA methylation in the promoter regions. Following in vitro methylation, the efficiency of each CpG methylation was confirmed by restriction enzyme digestion (Supplementary Figure S1). Figure 1 demonstrates that higher DNA methylation resulted in lower luciferase expression, reflecting a negative correlation between DNA methylation and gene expression. In addition, the activities of M. HhaI‐methylated and M. HapII‐methylated promoters were almost the same as the M. SssI‐methylated promoter. This implied that partial CpG methylation could silence *S100A6* expression and that M. HhaI‐methylated or M. HapII CpG at the *S100A6* promoter may play a vital role in modulating *S100A6* promoter activity.

**FIGURE 2:**
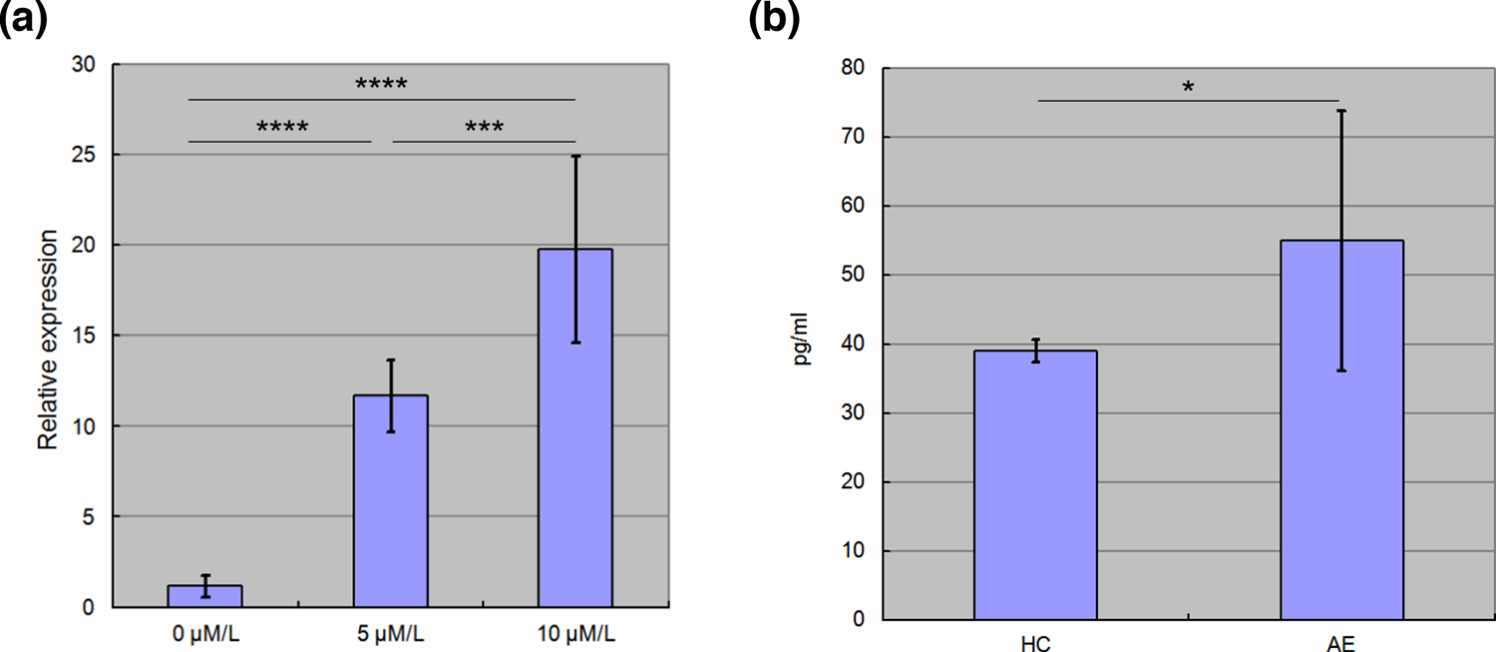
The results of 5‐azacytidine treatment and ELISA. (a) We had HL‐60 cells treated with 5‐azacytidine for 96 h, followed by a qPCR assay to examine S100A6. mRNA level was quantified with 2^−ΔΔCt^ by using 18S as an internal control. 5‐azacytidine treatment significantly enhanced the expression of S100A6 gene in a dosage‐dependent manner (*N* = 9, three replications * three rounds of assay). (b) Autoimmune encephalitis (AE) serum contained a significantly higher concentration of S100A6 protein than healthy control (HC) serum did (*N* = 8).

In addition to the methylation promoter assay, we also conducted 5‐azacytidine treatment and qPCR assays. As shown in Figure 2a, compared with the control set (non‐treatment), 5‐azacytidine treatment significantly induced higher S100A6 mRNA expression levels in HL‐60 cells in a dosage‐dependent manner. Taken together, the in vivo findings of our previous study (Tsai et al., 2019) and the in vitro findings in the present studies (Figures 1 and 2a) confirmed that the gene expression of *S100A6* was indeed regulated by DNA methylation.

We also conducted ELISA to measure the protein level of S100A6 in subject serum samples. As shown in Figure 2b, AE serum had a significantly higher S100A6 level than HC serum.

### 3.3 S100A6 enhanced adhesion between B lymphocytes and BBB endothelial cells

**FIGURE 3:**
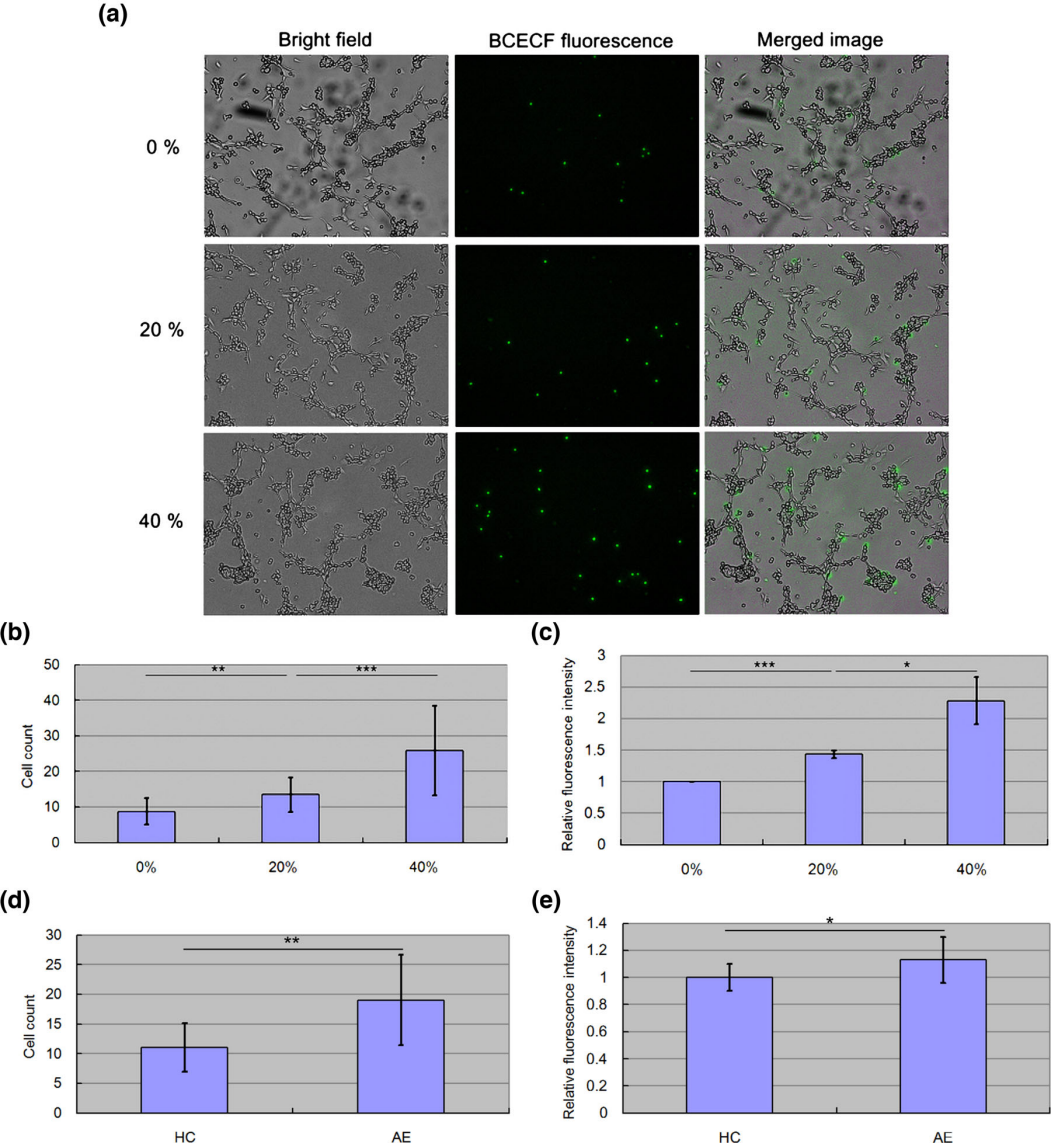
The results of the adhesion assay. Toledo cells were treated with different concentrations of S100A6 recombinant proteins, 0%, 20%, or 40% of the average concentrations in the AE patients. Toledo cells carrying green fluorescence were seeded on plates that had been coated with hCMECs in advance. After 2 h of co‐culture, the plates were washed with buffer to remove the Toledo cells that had not adhered to the hCMEC layer. More Toledo cells adhering to the hCMEC layer denoted higher adhesion ability. (a) The bright field and fluorescence columns illustrate the positions of hCMECs and Toledo cells, respectively. (b, c) An increase in S100A6 concentration caused a significant increase in adhesion ability (*N* = 3). (d, e) AE serum also caused a significant increase in adhesion ability than the control serum (*N* = 3).

Leukocyte infiltration is initiated by leukocyte rolling in the vessel lumen, followed by adhesion to vascular endothelial cells and further penetration of the endothelial barrier. In our previous study, we concluded that S100A6 promoted B lymphocyte penetration through the BBB (Tsai et al., 2019). Therefore, we hypothesized that S100A6 may enhance the initial adhesion between B lymphocytes and BBB endothelial cells. To test this hypothesis, we labeled Toledo cells (B lymphocyte line) with green fluorescence to recognize the Toledo cells adhering to hCMEC cells (BBB endothelial cell line). Figure 3a illustrates the positions of Toledo cells (the middle column, green fluorescence) and hCMECs (the left column, bright field) within the plates. As more S100A6 recombinant protein was applied, more Toledo cells adhered to the hCMEC layer after washing with buffer. For qualitative analysis, the number of Toledo cells in the plates was counted. Figure 3b demonstrates that significantly more Toledo cells adhered to the hCMEC layer as the applied concentration of S100A6 increased. In addition to cell count, quantification of the intensities of green fluorescence emitted from each plate also suggested that S100A6 enhanced adhesion between B lymphocytes and BBB endothelial cells (Figure 3c). Since AE patients had a significantly higher S100A6 serum level than HC subjects, we wonder whether serum treatment leads to similar results. Therefore, we also had Toledo cells treated with 20% serum from HC subjects or AE patients. Consistent with the results in Figure 3b,c, AE serum treatment also enhanced adhesion between B lymphocytes and BBB endothelial cells (Figure 3d,e).

### 3.4 The promoted B lymphocyte infiltration could be attenuated with the treatment of S100A6‐specific antibodies

**FIGURE 4:**
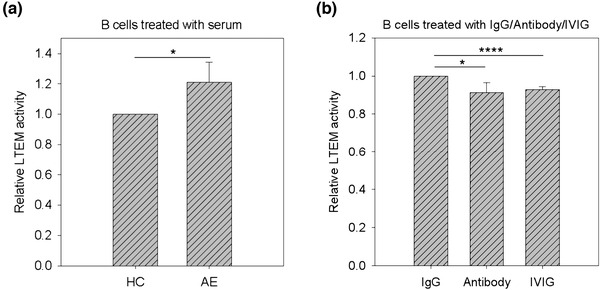
The results of leukocyte transendothelial migration (LTEM) assay of S100A6 antibody attenuation. We first treated pooled serum (20% serum) of the AE patients to mimic the disease, followed by S100A6 attenuation. (a) Compared with the serum from HC subjects (20% HC serum treatment), 20% AE serum treatment significantly enhanced the LTEM activity of Toledo cells (*N* = 3). (b) Compared with IgG control (AE serum + IgG), S100A6 antibodies (AE serum + antibody) attenuated the LTEM activity promoted by AE serum, resulting in an effect similar to human immunoglobulin (AE serum+IVIG; *N* = 3).

We previously demonstrated that S100A6 promoted B lymphocyte infiltration through the BBB endothelial layer using LTEM assays (Tsai et al., 2019). Based on this finding, we further investigated whether S100A6 could be a target to reduce B lymphocyte infiltration. We treated Toledo cells with pooled patient serum (pooling the serum samples from the 10 enrolled subjects) to mimic the disease, followed by LTEM assays. As shown in Figure 4a, compared with serum from the HC subjects, AE serum significantly enhanced the LTEM activity of Toledo cells and promoted their infiltration. AE serum‐treated Toledo cells were then treated with IgG control, S100A6 antibodies, or IVIG(a clinical treatment for AE). The LTEM assays showed that S100A6 antibodies and IVIG significantly reduced the LTEM infiltration activity of Toledo cells compared to IgG control (Figure 4b).

## 4 DISCUSSION

Combining the results of the current and our previous study (Tsai et al., 2019), we demonstrated that the difference in hypomethylation at the promotor regions of *S100A6* between HCs and AE patients resulted in the increased expression of S100A6. In addition, S100A6 enhanced the adhesion of B lymphocytes on BBB endothelial cells, which promoted the subsequent infiltration of B lymphocytes through the BBB. Moreover, we found that B lymphocyte infiltration through the BBB could be attenuated by S100A6 antibodies.

S100A6 is a member of the S100 protein family (Donato et al., 2017), which in turn belongs to the Ca^2+^‐binding protein family that acts as intracellular regulators and extracellular signaling proteins (Donato et al., 2013). S100A6 has been suggested to be involved in cell‐cycle progression (Matsuzawa & Reed, 2001), cellular response to stress(Spiechowicz et al., 2007), cytoskeleton dynamics (Golitsina et al., 1996), and binding to the transmembrane receptor for advanced glycation end products (RAGE; Leclerc et al., 2007). However, it remains unclear how S100A6 participates in promoting B lymphocyte infiltration in AE. There is evidence showing that S100A6 can change cellular motility, and knocking out S100A6 from NIH‐3T3 fibroblastic cells was shown to cause reorganization of the actin cytoskeleton thereby impairing cell adhesion and migration (Slomnicki & Lesniak, 2010). In previous studies of cancer cells, the effect of S100A6 on motility either facilitated or inhibited cellular migration (Luu et al., 2005; Nedjadi et al., 2009). In the present study, we found that the adhesion of B lymphocytes on the BBB endothelial layer and infiltration of B lymphocytes through the LTEM were all enhanced by applying S100A6. This suggests that S100A6 changes the motility of B lymphocytes, and further studies are warranted to investigate the underlying mechanism of this phenomenon.

Hypomethylation of the promotor region of *S100A6* has also been found in rheumatic arthritis (Svendsen et al., 2016). An altered gene expression pattern can serve as a potential marker of the activity of an autoimmune disease. This has been demonstrated by using interferon‐regulated chemokine genes to predict flare‐ups in patients with systemic lupus erythematosus. It was first shown by the upregulated expression of interferon in these patients (Baechler et al., 2003) and subsequently in another study that confirmed that some interferon(IFN)‐regulated chemokines could predict disease activity (Bauer et al., 2009). Few studies have investigated epigenetic control in AE, however, a similar epigenetic mechanism may also be present in AE. In our previous study, hypomethylation of three individual CpG sites within the *S100A6* promoter region was significantly negatively correlated with *S100A6* expression in AE patients (Tsai et al., 2019). In the present study, we further confirmed that promoter methylation inhibited the expression of *S100A6* in an in vitro methylation experiment. This finding suggests that promoter methylation can silence *S100A6* expression. Our results partially explain the epigenetic regulation related to AE. Further studies may lead to a clearer picture of the epigenetic process of AE and a better understanding of the disease mechanism, which may then be used to develop a prediction model for the activity of AE.

S100A6 interacts with a wide range of protein ligands, some of which are related to immunological response (Filipek & Lesniak, 2018), including RAGE (Filipek & Lesniak, 2018; Xie et al., 2013). S100A6 has been shown to be increased in the serum of patients with systemic sclerosis, and this has been correlated with disease activity (Balanescu et al., 2021). Integrin, a transmembrane receptor that facilitates cell–cell and cell–extracellular matrix adhesion, has also been shown to interact with S100A6 (Jurewicz et al., 2014). The expression of integrin in circulating human B cells has been shown to assist their entry into the simulated BBB barrier (Alter et al., 2003). In this study, we showed that by blocking the effect of S100A6 with its antibody, the infiltration of B lymphocytes through the LTEM was attenuated. It is unclear whether S100A6 acts through the RAGE or integrin pathway to facilitate the penetration effect of B lymphocytes through LTEM, and further studies are warranted to clarify this issue. Our observations suggest that S100A6 may be a new therapeutic target or marker for AE.

The major limitation of this study is that LTEM simulated only the endothelial layer of the BBB. The integrity of the BBB is maintained by a multicellular structure including pericytes, vascular smooth muscle cells, various glial cells, and the basement membrane (Sweeney et al., 2019). Importantly, the basement membrane limits the transmigration of leukocytes (Sixt et al., 2001); therefore, penetrating the endothelial layer does not guarantee the initiation of the inflammatory process. Moreover, neural tissue by itself also provides an anti‐inflammatory environment that hinders the survival of immune cells (Irani et al., 1996; Strle et al., 2001). The complex interactions between the BBB and its surrounding tissues are difficult to imitate; however, we demonstrated the regulatory role of S100A6 in the invasion of the BBB endothelial layer, which has been postulated to be a potential route for entering aberrant immune cells (Platt et al., 2017). The participation of other inflammatory mediators is also likely to be involved in damaging the BBB, and further studies are warranted to investigate this issue.

## AUTHOR CONTRIBUTIONS

All authors have read and approved the final manuscript. Conceptualization: Chih‐Hsiang Lin, Meng‐Han Tsai, and Sung‐Chou Li, Methodology: Kuo‐Wang Tsai, Sung‐Chou Li, Ming‐Hong Lin, Yuyu Lin, Pei‐Hsien Lin, and Meng‐Han Tsai. Formal analysis: Sung‐Chou Li, Ming‐Hong Lin, Yuyu Lin, and Pei‐Hsien Lin. Investigation: Chih‐Hsiang Lin, Chen‐Jui Ho, and Yan‐Tin Lu. Writing–original draft preparation: Chih‐Hsiang Lin and Sung‐Chou Li. Writing–review and editing: Chih‐Hsiang Lin and Meng‐Han Tsai. Supervision: Meng‐Han Tsai.

## CONFLICTS OF INTEREST

The authors declare that there is no conflict of interest.

### PEER REVIEW

The peer review history for this article is available at https://publons.com/publon/10.1002/brb3.2897.

## Supporting information

FIGURE S1 Confirmation of methylation efficiency. pGL‐S100A6 promoter vectors were methylated by M. SssI, M. HhaI, or M. HpaII. After in vitro methylation, the efficiency of each methylating plasmid was confirmed by restriction enzyme (HhaI and HpaII) digestion.

## Data Availability

The datasets generated and analyzed during the current study cannot be made openly due to ethical concerns but are available from the corresponding author upon reasonable request.
