# Supplementary figures and images for: S100A6 participates in initiation of autoimmune encephalitis and is under epigenetic control
Source: Brain Behav. 2023 Feb 7;13(3):e2897. doi: 10.1002/brb3.2897 (PMC10013942; doi:10.1002/brb3.2897)

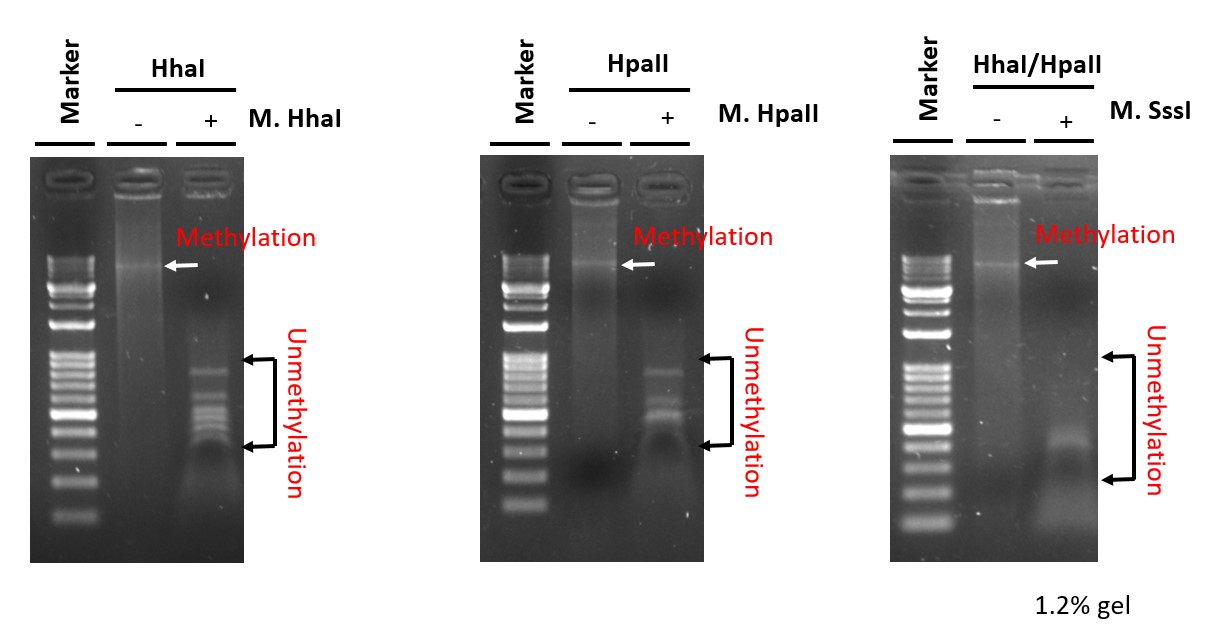

Supplement: Supplementary file 1 — FIGURE S1 Confirmation of methylation efficiency. pGL‐S100A6 promoter vectors were methylated by M. SssI, M. HhaI, or M. HpaII. After in vitro methylation, the efficiency of each methylating plasmid was confirmed by restriction enzyme (HhaI and HpaII) digestion. [file BRB3-13-e2897-s001.jpg]
